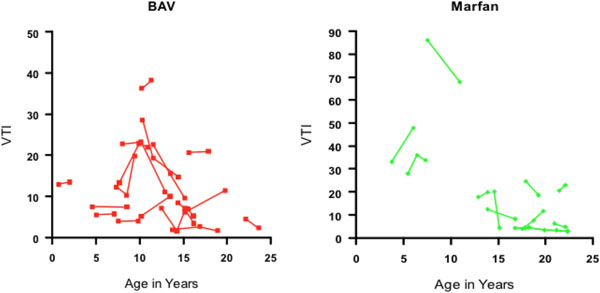# Arterial tortuosity and change with age in young patients with aortopathy

**DOI:** 10.1186/1532-429X-17-S1-P403

**Published:** 2015-02-03

**Authors:** Shaine A Morris, William A Payne, Sarah Sami, Yunfei Wang, Scott A LeMaire, Jon E Tyson, Rajesh Krishnamurthy, Dianna M Milewicz

**Affiliations:** 1 Pediatric Cardiology, Texas Children's Hospital, Houston, TX, USA; 2 Pediatrics, Baylor College of Medicine, Houston, TX, USA; 3 Cardiovascular Surgery, Texas Heart Institute, Houston, TX, USA; 4 Cardiovascular Surgery, Baylor College of Medicine, Houston, TX, USA; 5 Pediatrics, University of Texas Medical School at Houston, Houston, TX, USA; 6 Radiology, Texas Children's Hospital, Houston, TX, USA; 7 Genetics, University of Texas Medical School at Houston, Houston, TX, USA

## Background

In pilot studies, increased vascular tortuosity is associated with adverse outcomes in children and young adults withheritable thoracic aortic disease, specifically Marfan syndrome (MFS) and Loeys Dietz syndrome (LDS). It is known that vertebral artery tortuosity is highly prevalent in older adults who do not have heritable thoracic aortic disease. We hypothesized that tortuosity increases with age in patients with aortic disease <50 years old.

## Methods

We included patients followed at our institution with a diagnosis of MFS, LDS, Turner syndrome (TS), bicuspid aortic valve, and non-specific connective tissue disorder, who have undergone at least 2 magnetic resonance angiograms including the entire vertebral artery a minimum of 1 year apart. In a blinded fashion, using a volume-rendered projection, each patient's vertebral artery tortuosity index (VTI) was calculated using the larger distance factor (% by which actual length exceeds straight-line length) of the two vertebral arteries. Using linear longitudinal regression analysis, we evaluated the change in VTI per year overall and by diagnostic category.

## Results

Forty patients with a total of 104 serial MRAs were included, including 18 with BAV, 11 with MFS, 3 with LDS, 5 with Turner syndrome, 2 with non-specific connective tissue disorders, and 1 with coarctation of the aorta. Patients with LDS had the highest median VTI (54, range 14-235), followed by MFS (median 15, range 3-86); the lowest VTIs were in BAV (median 10, range 2-38) and TS (median 5, range 0-19). Overall, among diagnostic groups, the youngest patients had the highest VTIs. Serial imaging demonstrated an overall trend of slowly decreasing VTI through childhood with a plateau after adolescence, with a mean change in VTI of -1.2 per year (p=0.01). Of the larger diagnostic groups, patients with Marfan syndrome had a slightly faster decline in VTI than BAV (-1.7 per year vs. -0.4 per year, p=0.03).

## Conclusions

Increased arterial tortuosity as measured by VTI is present in conditions associated with higher risk of aortic dissection. Contrary to our hypothesis, VTI slightly decreased with age into the adolescent period. Further investigation need to be performed to demonstrate potential etiologies of this, which may include increases in height, medical therapy, or change in vascular signaling with age.

## Funding

Baylor College of Medicine Cardiovascular Research Institute Pilot Award and NHLBI 1R21HL121630-01A1.

**Figure 1:**